# Evaluation of Chemical Mechanical Polishing-Based Surface Modification on 3D Dental Implants Compared to Alternative Methods

**DOI:** 10.3390/ma11112286

**Published:** 2018-11-15

**Authors:** Riaid Alsaeedi, Z. Ozdemir

**Affiliations:** 1 Mechanical Engineering Department, Ozyegin University, Istanbul 34794, Turkey; Zeynepozdemir34@gmail.com; 2 Mechanical Engineering Department, University of Diyala, Diyala 32001, Iraq

**Keywords:** chemical mechanical polishing, biocompatibility, surface structuring

## Abstract

Chemical mechanical polishing (CMP) has been introduced in previous studies as a synergistic technique to modify the surface chemistry and topography of titanium-based implants to control their biocompatibility. In this study, the effectiveness of CMP implementation on titanium-based implant surface modification was compared to machined implants, such as baseline and etching and biphasic calcium phosphate (BCP) particle-based sand blasting treatments, in terms of the surface chemical and mechanical performance. Initially, a lab-scale 3D CMP technique was developed and optimized on commercial dental implant samples. The mechanical competitiveness of the dental implants treated with the selected methods was examined with the Vickers microhardness test as well as pull-out force and removal torque force measurements. Furthermore, the surface structures were quantified through evaluation of the arithmetic mean roughness parameter (Ra). Subsequently, the surface chemistry changes on the treated implants were studied as wettability by contact angle measurement, and surface passivation was evaluated through electrochemical methods. In each evaluation, the CMP treated samples were observed to perform equal or better than the baseline machined implants as well as the current method of choice, the BCP treatment. The ability to control the surface topography and chemistry simultaneously by the use of CMP technique is believed to be the motivation for its adaptation for the modification of implant surfaces in the near future.

## 1. Introduction

Implant therapy has become widely accepted in the community of prosthetic surgery, especially in dentistry, making it a preferred alternative for rehabilitation of partially or totally edentulous patients. Titanium and its alloys (most commonly Ti–6Al–4V) are widely used biomaterials in prostheses, cardiovascular devices, and fracture fixation due to their high biocompatibility, low density, high strength-to-weight ratio, and superior corrosion resistance due to fast passivation and growth of their protective native oxide film [1]. Compared with other metallic biomaterials, titanium also has the advantage of having a greater tendency to osseointegrate, which is the most important characteristic for long-term implant retention. The limited reaction of titanium with the surrounding tissues is mainly driven by surface passivation due to the titanium dioxide (TiO_2_) film formation that is known to be in the nanometer range of thickness (3–10 nm). The self-protective nature of the titanium oxide improves corrosion resistance and provides improved biocompatibility to the titanium implant surface [2,3].

In addition to the biocompatibility, the metallic prostheses also need to have mechanical compatibility with the bone tissue they replace. This can be achieved when the selected metal has an elastic modulus close to the bone, a favorable strength-to-density ratio and high resistance to mechanical failure. While the short- and long-term osseointegration performances of an implant are typically evaluated as functions of the material selection, surface topography and chemical nature, the mechanical performance of an implant is also critical and is generally correlated to the dimensional design and surface treatment driven hardness change, as well as the surface degradation prevention through improved corrosion resistance.

In general, research efforts on metallic biomaterials have been directed towards the development of superficial modifications which improve their mechanical as well as biological properties in addition to improving their resistance to wear, corrosion, and fatigue [4]. An important aspect to take into account when modifying the surface of a material is to consider the specific requirements of the clinical application for which the implant is designed. As an example, some implants are used as temporary placements and they require minimal interaction with bone tissue to facilitate their removal after their service time. On the other hand the permanent implant materials in the area of attachment to the bone of uncemented prostheses should interact with the tissue promoting osteointegration [5]. In this case, the long-term efficacy of the implant depends, to a large extent, on the first phase of interaction with the osteoforming cells as well as on the implant’s capacity to facilitate cell proliferation, differentiation, and mineralization on the surface.

Various superficial modifications have been investigated in recent years to control the adhesion of bone cells to the implant material, leading to the development of techniques and methodologies aimed at modifying the chemical or topographical properties of the conventional biomaterial surfaces [6]. The modifications in the topography of a metallic biomaterial allow anchorage to the bone tissue, reduce the osteointegration time and obtaining a greater transmission of occlusal mechanical loads between the bone and the implant [7]. Efforts to improve the osseointegration are typically approached by creating rough surfaces that increase the surface area available for the bone-to-implant binding (mechanical blockage) and optimize fixation and stability [8]. Few studies have been devoted to quantifying the biomechanical behavior simultaneously as a function of the topographic changes on the surface of the metallic bioimplants [9].

In the present study, a systematic approach is taken by considering the machined dental implants as the baseline samples and comparing the chemical etching, biphasic calcium phosphate (BCP) sand blasting and chemical mechanical polishing (CMP) surface treatment methods as alternative techniques to evaluate their impact on implant surface topography, surface chemistry as well as the mechanical performance. All the dental implants are produced by precise machining techniques with a given design of the screws. Acid treatment is performed to detach any residual metal particles from the surfaces of the implant by immersing them in an acidic solution. However, it also leads to surface corrosion and tends to leave areas of higher corrosion in the peaks and valleys of the grooves designed by the surface fabrication. The advantage of this method lies in the fact that it enables control of the degree of porosity on the surface while cleaning the unwanted impurities and particles from the implant surface [10]. The acid etching method has been observed to increase the cell adhesion and bone formation, thus enhancing the osseointegration due to the increase of the bonding of the surface for luting agents [11].

Another surface treatment that is typically used for implant surface structuring is sandblasting, which is a jet of particles aimed towards the implant surface with a durability greater than that of titanium, such as aluminum oxide, titanium oxide, silica, hydroxyapatite, and beta-tricalcium phosphate (BCP). BCP is also known as RBM (Resorbable Blast Media) treatment [12]. These particles generate a series of irregular surface indents, which are called macro-retentions. However, this method has the drawbacks of having a nonhomogeneous surface topography, the attachment of residual particles to the surface, and a limited ability to control surface roughness precisely [13].

In addition to the etching and sand blasting techniques, there are other commonly implemented surface treatments that alter the surface composition of the bioimplant materials, such as thermal oxidation, ionic implantation, anodizing, and acid or alkaline treatments. The main purpose of these treatments is to improve the resistance to wear and corrosion as well as to improve the biocompatibility of the implant surface [14,15]. As an example, the thermal oxidation of the Ti–6Al–4V alloy was observed to result in the formation of an oxide layer that decreased the ion release and improved the adhesion and proliferation of the bone forming osteoblastic cells [14]. In addition, anodization treatments on the same alloy induced an increase in the activity of alkaline phosphatase (the enzyme required for the formation of the bone) in the osteoblast cells [16]. While these surface treatments lead to changes in the chemical composition of the substrate, the deposition of coatings allows the introduction of new surface compositions that are different from the base material. Nevertheless, anodization is an effective, simple, inexpensive, and conformal type of nanopatterning which makes it applicable for use on the curved surfaces of dental implants. Furthermore, the new trend of using acid electrolyte anodization provides a continuous, homogeneous, and porous coating film which can be used as a bioactive drug loading cavity [17]. Anodization is one of the few surface treatments that can achieve a coherent set of requirements in terms of (i) decontaminating the implant surface from the organic and inorganic impurities that could affect the formation of the oxide layer [18], (ii) avoiding ion release to the surrounding hard and soft tissues by (iii) increasing the corrosion resistance, (iv) improving the wear resistance due to the increase in hardness of the surface of the dental implant, and (v) increasing the biocompatibility and bone formation with the possibility of adding different chemical species, like Mg which is essential for the absorption of calcium minerals in bone cells [17].

Chemical mechanical polishing on titanium bioimplants was recently introduced by Z. Ozdemir, A. Ozdemir, and G.B. Basim as an alternative technique for bioimplant surface structuring [3]. CMP can induce the synergistic effect of surface nanostructuring while altering the surface chemistry to minimize surface corrosion and ion dissolution through the promotion of formation of a self-protective surface oxide film [2]. Preliminarily, the CMP technique was applied through hand polishing on 3D dental implant samples, and biocompatibility performances were evaluated in terms of cell toxicity, cell growth, and limited bacterial attachment [3]. Since the CMP technique provides spontaneous chemical activation of the implant surface through the use of slurry chemicals, it is an effective surface treatment for Ti-based implants to render them more biocompatible. The treatment was shown to be benign for the cell viability and allowed the control of cell attachment/detachment through the degree surface topography. The bio-implants success or failure was reported on the basis of biocompatibility without detailed study on the mechanical performance. 

In the present study, the 3D CMP technique was automated at the lab scale to provide a unified set of features on the dental implants by controlling the following process variables: down force, rotational velocity of the sample and the polishing pad, selected pad type, process time, particle characteristics, slurry chemistry, and substrate material type. Beyond the optimization of the CMP technique, the chemical and mechanical properties of the implants treated with CMP were compared to those produced by baseline machining, BCP sand blasting and etching implementations. Moreover, since this treatment is unprecedented for the surface structuring of bioimplants it was important to compare the results with another surface treatment method with the same path of promotion for titanium oxide layer growth on implant surfaces. Likewise, control of the oxide layer thickness and its porosity were essential approaches in terms of enhancing the wear prevention and biocompatibility. So, anodization is the closest and the most effective surface structuring method to be compared with, through which it is possible to change not only the microtopography but also the chemistry of the implant surface. The most successful anodized surface in the market is TiUnit (Nobel Biocare) which has had a 97.69% survival rate over the last 10 years [19]. In recent work, the obtained results from CMP treatment were compared to that of TiUnit in terms of the wettability, surface roughness, corrosion behavior, Vickers microhardness, and removal torque.

## 2. Materials and Methods

### 2.1. Dental Implant Treatment and CMP Equipment Set-Up

**Figure 1:**
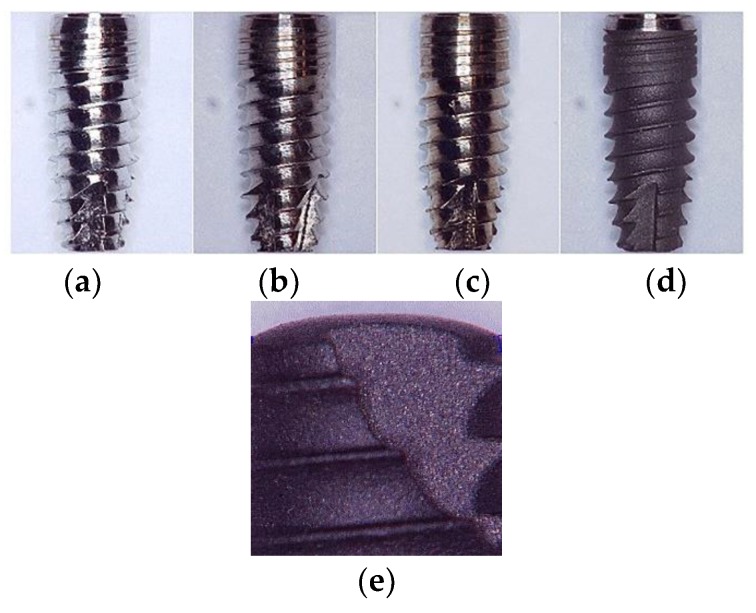
Optical images (50× magnification) of (**a**) baseline (machined), (**b**) chemical mechanical polishing (CMP), (**c**) etched, and (**d**) BCP treated dental implants, (**e**) micrograph of a biphasic calcium phosphate (BCP) dental implant.

Titanium dental implants with diameters of 3.3 mm and heights of 8 mm were provided by MODE Medical Company in Istanbul, Turkey [20]. Three-dimensional (3D) dental implant samples are illustrated in Figure 1 as a function of the applied surface treatment, and Figure 1b–d illustrate the CMP, etch, and BCP treated implants, respectively. Figure 1a shows the precisely shaped baseline implant after machining before any surface modification was applied. Figure 1b illustrates the CMP treated sample with a silica slurry with an average particle size of 20 nm and a pH of 3 that was obtained from BASF, Germany. The slurry pH was adjusted by NaOH to pH 9, and hydrogen peroxide (Sigma Aldrich with purity 34.5–36.5 wt%) was used as an oxidizer at a concentration of 0.1 M. Slurry solid loading was varied at 3, 5, and 10 wt% to modify the pressure per particle and hence, the surface microstructure.

**Figure 2:**
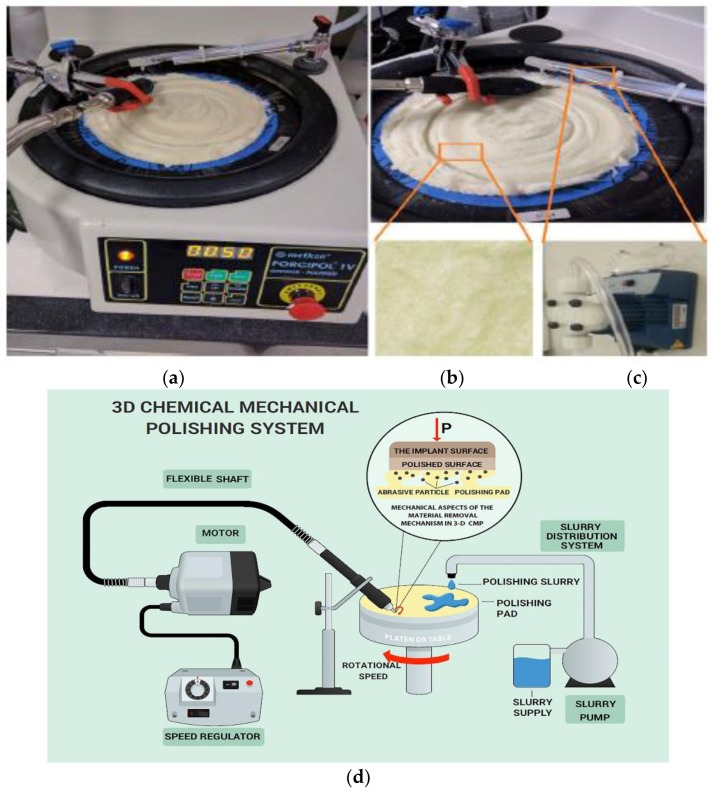
(**a**) The set-up developed in the lab to implement the 3D CMP on the dental implants, (**b**) synthetic wool pad, (**c**) solenoid dosing pump (**d**) The 3D CMP set-up configuration.

The 3D CMP was performed by using a lapping tool (Metkon, Forcipol 1 V) to carry the polishing pad with different rotational velocities of 50, 60, 70, and 80 rpm, as illustrated in Figure 2a. CMP slurries with 3, 5 and 10 wt% solid concentrations were flown at a flow rate of 40 mL/min by using a SEKO solenoid dosing pump on a synthetic wool polishing pad (Dekor 1056 20 cm pure wool), as shown in Figure 2b,c, respectively. The chemically etched samples were prepared by dipping the implants in hydrogen peroxide (37 wt%) for 30 min, as illustrated in Figure 1c. The BCP surface treated samples were provided by the Mode Medical Company and prepared by jetting calcium derivatives (biphasic calcium phosphate), as shown in Figure 1d. Figure 2d illustrates the 3D CMP process scheme that was designed in the lab.

All the samples were cleaned in an ultrasonic bath with ethanol for 10 min, rinsed with Deionized water and then dried by blowing air before they were characterized.

#### Presentation of the Lab-Scale Setup

The developed lab-scale technique proposed in this study consisted of equipment that was deliberately grouped to meet the purpose for which it was combined. The most important component in CMP is the polishing pad, and its characteristics must be provided in detail in terms of the structure and materials [21]. The design of the polishing pad utilized in this work consisted of a surface layer of flexible brush-like microfibers with the ability to efficiently carry the nanoscale abrasives in the slurry independent of the sample down pressure. The low sample down pressure associated with the predefined solid loading and oxidizer concentration, allowed a good surface uniformity to be produced effectively while limiting the material removal rate [22]. Furthermore, the correlated specifications of the polishing pad included (i) a fleecy texture with a large number of microfibers to increase the abrasive carrying capacity and, as a result, increase the contact frequency between the pad and the sample; and (ii) a pad basal soft layer to effectively propose a better slurry delivery, conform to the implant surface variation, and achieve good surface uniformity. In addition, the rotational motion of the platen to which the pad is attached was needed to ensure and maintain a good contact frequency with the surface being polished. Moreover, the solenoid dosing pump had a preset feature to feed the slurry in a specific period and quantity determined by the operator in order to keep the slurry delivery within the desired limits. Finally, the adjusted rpm of the sample holder which was attached to a flexible shaft connected to an electrical motor gave an axial rotating motion (spinning motion) to the sample on the rotating polishing pad connected with the presence of the slurry to ensure a uniform CMP treatment.

### 2.2. Experimental Methods

#### 2.2.1. Chemical Mechanical Polishing Application

The sample holder was attached to a flexible shaft connected to an electrical motor, and the rotational speed was controlled by an rpm regulator. CMP tests were carried out at different rpm values (88, 260, 450, and 740 rpm). The polishing pad was replaced with a new one after polishing a given set of samples to maintain a certain roughness level to ensure process performance stability through standard compressibility of the pad to provide efficient slurry delivery to the surface. Figure 2a shows the CMP set-up developed in the lab to implement the 3D CMP onto the dental implants. In the 3D CMP implementation on the 3D samples, the down force was maintained at a constant level, and only the angular velocities of the pad and the samples were varied.

The CMP treated samples were characterized by their surface topography (surface roughness), wettability, and material removal rates as functions of CMP input variables. The chemical mechanical polishing was run for 10 min in the presence of 0.1 M H_2_O_2_ at slurry solid loadings of 3, 5, and 10 wt%.

#### 2.2.2. Material Removal Rate (MRR) Determination

The material removal rates obtained with the various CMP treatments were calculated by weighing the samples pre and post CMP with a Swiss Made ES125SM model high precision scientific balance (five digits after the decimal point, 0.01 mg accuracy). In order to determine the oxidizer concentration that gave the lowest material removal rate and hence, the minimal number of dimensional changes on the samples, oxidizer was added at concentrations of 0.001, 0.01, 0.075, 0.1, 0.3, and 0.5 M. In addition, five baseline samples were polished without addition of H_2_O_2_ to investigate the influence of the presence of oxidizer on the material removal rate.

#### 2.2.3. Surface Roughness Evaluations

Since the surface roughness of an implant is a determining factor of its surface quality [23], the surface roughness measurements of the 3D samples were performed with a High-accuracy Surftest SJ-400 Mitutoyo profilometer. The pre and post CMP surface treatment values of the surface roughness (Ra) were recorded as an average of three measurements taken on the 3D samples. To determine the improvement of implant performance in accordance with the literature findings, the main strategy followed was to obtain rough surfaces of Ti implants in a controlled manner by selecting the variables for the CMP process to give the desired surface topographical results [24].

#### 2.2.4. Wettability Characterization

All the implant samples were characterized pre and post different surface treatment applications for their wettability. The sessile drop method was used to determine the surface wettability through contact angle measurements on the 3D sample surfaces by using a KSV ATTENSION Theta LiteOptic Contact Angle Goniometer (using DI water as the liquid phase). The size of the drop was maintained at ~140 µm. To investigate the uniformity of the surface processing, five drops were measured on each sample on a pre-selected region where the thread pitch was the same. The images of the drops on the samples were recorded by a high-speed camera, and the contact angles were calculated with the help of image analysis software.

#### 2.2.5. Electrochemical Corrosion Assessment

Three-dimensional Ti surface treated implant samples with a density of 4.5 g/cm^3^ were subjected to potentiodynamic scans that were performed in DI water at pH 6.5. The scans were collected at a range from −5 V to 6 V with a scan rate of 10 mV/s and a step of 1 mV for each point. Tafel data were calculated for each scan. $I_{corr}$ and $E_{corr}$ were used to calculate the corrosion rate of the software.

#### 2.2.6. Biomechanical Evaluations

Biomechanical evaluations of different surface treatments were performed by implementing three approaches: (i) the implant pull-out test, (ii) the removal torque test, and (iii) surface hardness measurements.

##### Pull-Out Test

The four groups of implants with different surfaces were evaluated to identify their primary levels of stability and to understand the effects of immediate loading on dental implants, an indicator of great significance to the guarantee of osseointegration [25]. The bone–implant interfacial debonding was evaluated to assess the implant primary stability. Throughout the pull-out strength measurements, the only variable was the surface treatment. After fixation, the dental implants were automatically and completely removed with a standardized extraction speed of 2 mm/min. The pull-out strength was documented, the peak of which was assumed to be the failure of the bone–implant interface.

**Figure 3:**
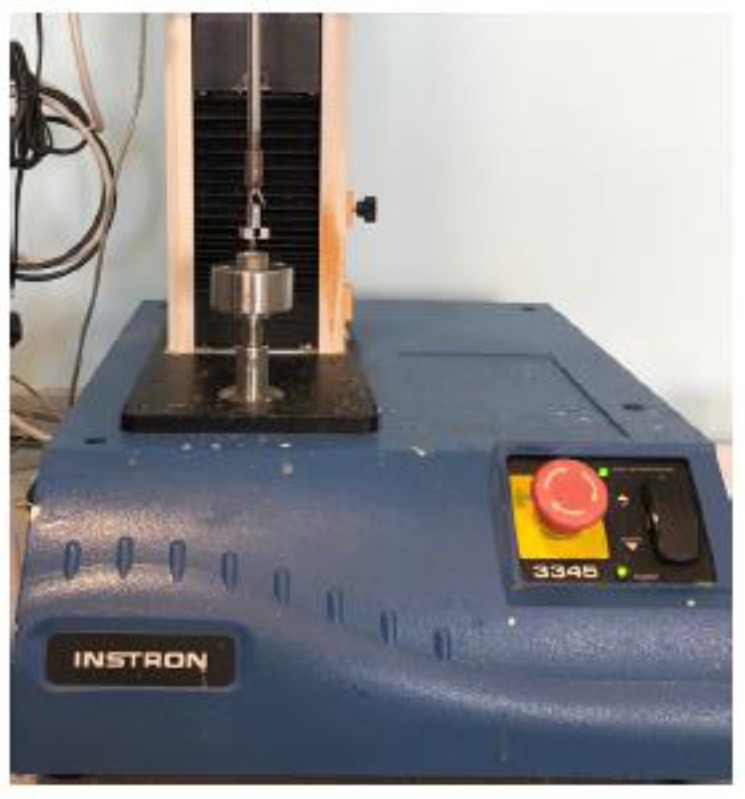
Pull-out test machine (Instron 3345 series).

The pull-out test is one of the most important assessments in the determination of the primary stability of an implant in bone tissue as a predictor of bone osseointegration. Instron 3345 series equipment was used for the pull-out test with a custom designed holder to fit the dental implants to the pull-out testing machine (Figure 3). Twenty implants measuring 8 mm in length were implanted and labeled into 4 groups, with each group being exposed to a different surface treatment. In this approach, to evaluate the implants’ primary stability, the surface topography was the only variable. After fixation, the dental implants were automatically removed with a standardized extraction speed of 2 mm/min, and the pull-out strength was documented.

##### Removal Torque Test

Numerous research works have seeked to understand the influence of the modification of titanium implant surfaces on the response of tissues [10]. For this, different treatments have been carried out with the aims of reducing the time necessary for osseointegration, increasing the initial biomechanical anchorage, and promoting bone formation. In the present work, the surfaces were analyzed and the necessary torque for the extraction of treated dental implants with four different treatments was evaluated. The results obtained allowed characterization of the surfaces and determination of the differences in the initial response. The implant with a combination of physical and chemical treatments showed a greater extraction torque.

**Figure 4:**
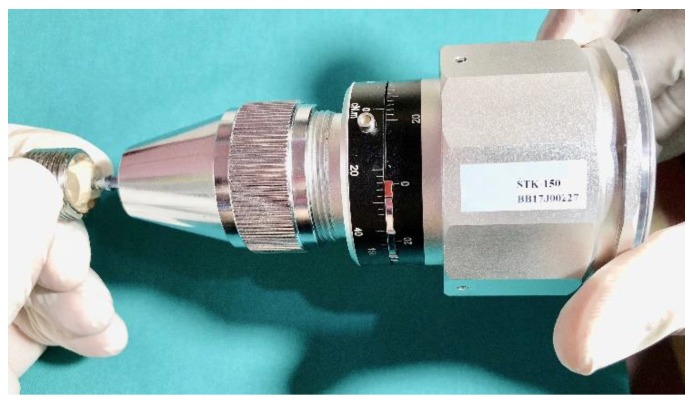
Extraction of implants from the bone using the torque calibrator (Sundoo STK-150cN.m Gauge).

The removal torque test is an assessment of bone quality and support where the minimum required level of primary stability can be predicted for immediate loading. The removal torque values were measured with a torque calibrator. A Sundoo STK-150cN gauge was positioned in the same direction as the implant axis to measure the removal torque at the time of final seating of the implant (Figure 4). Thirty-two implants were tested, with 16 of them measuring 8 mm and 16 of them measuring 10 mm in length; these were implanted into bone. The implants with 8 mm length were divided into 4 groups with each group receiving a different surface treatment. The implants of 10 mm length and 4.1 diameter were also grouped based on their surface treatments. The length of the implant and the amount of bone-to-implant contact were the determinants for the interface strength.

##### Microhardness Test

The Future Tech, FM-300e Vickers hardness tester was utilized for the samples treated by the 4 selected techniques. Since the surface structuring methodology and surface roughness affect the implant surface hardness simultaneously, it is critical to measure hardness to evaluate the implant’s long-term mechanical integrity. Microhardness tests were conducted to provide a reference for comparison between the four different surface treatments. Using a microhardness tester (Future Tech, FM-300e, Kanagawa, Japan) with a Vickers pyramidal tip indenter, a 1000 gm load was applied to previously surface treated samples of each of the four surface treatment methods over a period of 10 s. Five indentations were made at different locations on the samples’ surfaces.

## 3. Results and Discussion

### 3.1. CMP Performance Evaluations

#### Effect of the H_2_O_2_ Concentration on the Material Removal Rate

**Figure 5:**
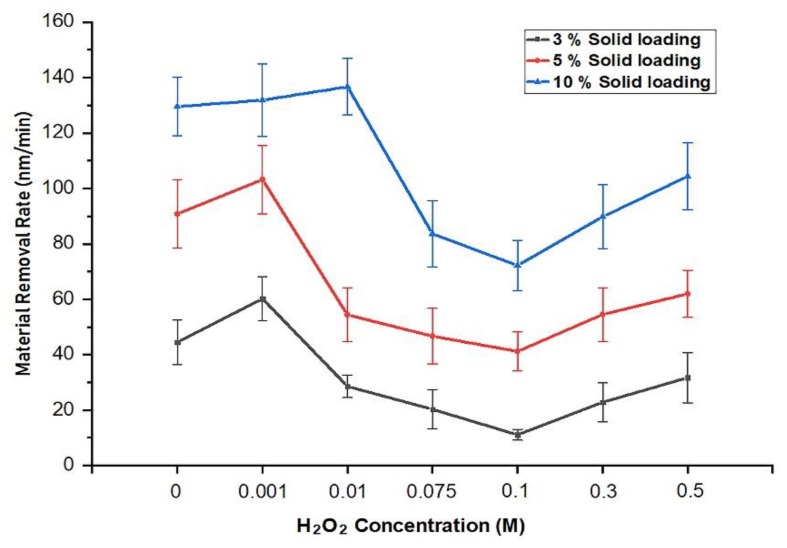
Material removal rates of Ti-based dental implants at different H_2_O_2_ concentrations and with a rotational speed of (88, 50) rpm for the sample and the pad respectively.

Figure 5 illustrates the CMP performances based on the material removal rate (MRR) measured for 3D CMP with the developed process setup at a constant pad-sample rpm. To detect the optimal H_2_O_2_ concentration and obtain the minimum MRR, seven different oxidizer concentrations were tested (0, 0.001, 0.01, 0.1, 0.2, 0.3, and 0.5 M) together with slurry solid loadings of 3, 5, and 10 wt%. Sixty-three samples were tested, with three samples for each test. It can be seen that the effects of slurry oxidizer concentration and slurry solid loading on the weight loss of the measured implant samples was very significant. The material removal rates decreased as the oxidizer concentration increased to a point where the morphology of the oxide film changed and the surface was passivated, that is, controlled by the surface chemical activity. On the other hand, at higher slurry solid loadings, MRR values tended to be higher as a result of an increase in mechanical activity provided by the slurry particles, which acted as mechanical cutting tools on the chemically activated surface.

Figure 5 summarizes the results of the CMP MRR findings, highlighting that the adequate H_2_O_2_ concentration for minimal MRR was 0.1 M for all solids loadings tested. The presence of oxidizer in the slurry solution helped to passivate the titanium surface by converting Ti to TiO_2_ [3,26].

**Figure 6:**
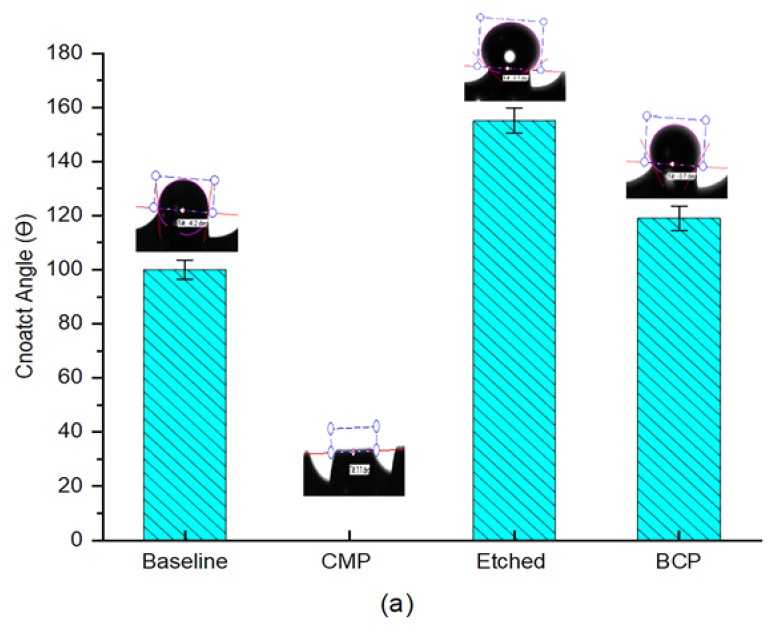

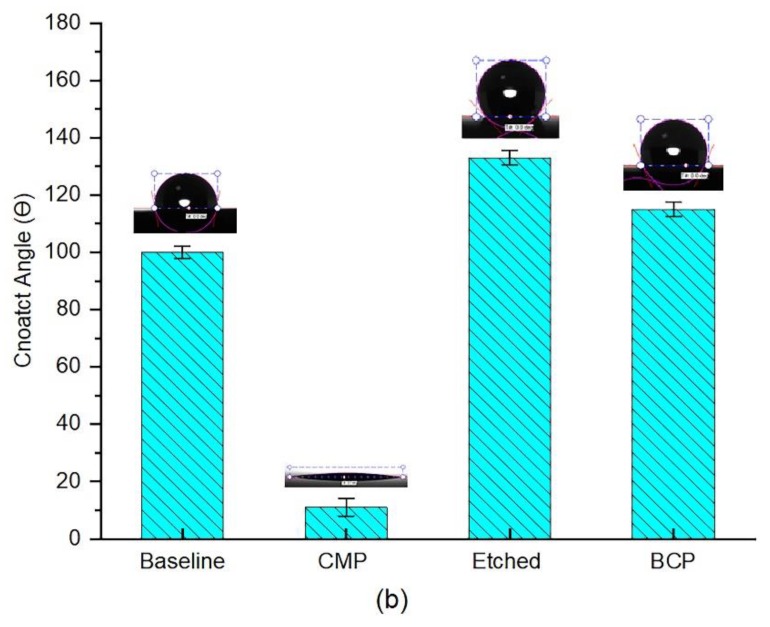
Surface wettability of the baseline and differently treated surfaces of (**a**) Ti-based implants, (**b**) Ti-based plates.

The passivating action assists the material removal action, because the oxide is more brittle than the titanium metal. Passivation also slows the removal rate due to the oxide layer acting as an etch stop layer that prevents the chemicals from etching the titanium surface. Based on the results presented in Figure 6, it can be concluded that the surface passivation continued up to an oxidizer concentration of 0.1 M, leading to a continuous reduction in MRR values. Beyond this concentration, the addition of the H_2_O_2_ seems to result in a porous and unprotective oxide layer that tends to increase the MRR values as a result of the combination of mechanical removal with the continuing chemical etching on the metals’ surfaces.

### 3.2. Wettability Evaluations

Figure 6a,b summarize the wettability behavior elucidated by the contact angle measurements taken with DI water droplets on the titanium-based implant and plate samples that were subjected to different surface treatments.

The relatively high contact angle measured for the baseline samples (100°) is believed to be dependent on both the surface topography and the chemical nature. According to the Cassie-Baxter model [27], when a water droplet stays on top of a solid texture with air trapped underneath, hydrophobic behavior can be observed, such as that observed with the machined samples. Since the surface topographic nature is irregular, it tends to trap air in between the water droplet and the implant surface. On the other hand, the sample treated with 3D CMP demonstrated full spreading of the water droplet. This is due to the fact that CMP treatment results in a refreshed surface being exposed to the water droplet with a greater number of dangling bonds, making the surface more hydrophilic with a smoother interface [28]. The implementation of 3D CMP with a slurry solid loading of 5 wt% resulted in a more homogeneous wetting regime and facilitated the absorption of DI water on the implant surface. The contact angle was reported to be 0°, although we know that this measurement was the result of insufficiency of the optical measurement of the contact angle on the threaded hydrophilic surfaces, where the liquid spreads and the contact line cannot be recognized.

On the other hand, the contact angle measured for different samples treated with BCP possessed a higher contact angle of 119°. The micrograph given in Figure 1e shows the rough surface which is the main reason behind the higher hydrophobicity of the BCP treated samples. Finally, chemical etched implants showed higher hydrophobicity with an average contact angle of 155°. The enhanced hydrophobicity is believed to have been caused by the presence of patterned morphology along with the low surface energy due to the long period of time required for etching (30 min dipping) in 37 wt% H_2_O_2_, despite the increased surface roughness values. In order to take the microstructure of the implant into account for this evaluation, control experiments were conducted on Ti-based plate samples that had been treated respectively. Figure 6b illustrates the results obtained from the contact angle test on the Ti-based plate samples. The wettability behavior of the plates was identical to that of the pretreated dental implants. The slight difference in CA values was due to the planar and cylindrical-threaded liquid interfaces that were used for the plates and dental implants respectively [29].

Meanwhile Manfro, Rafael et al. identified a 10% improvement in the wettability capacity of anodized dental implants compared to the surface obtained by the etching treatment [30]. Clearly, anodizing treatment diminishes the surface energy and increases the surface hydrophilicity, as is the shown by this study.

### 3.3. Effect of the Slurry Solid Loading on the Surface Roughness

**Figure 7:**
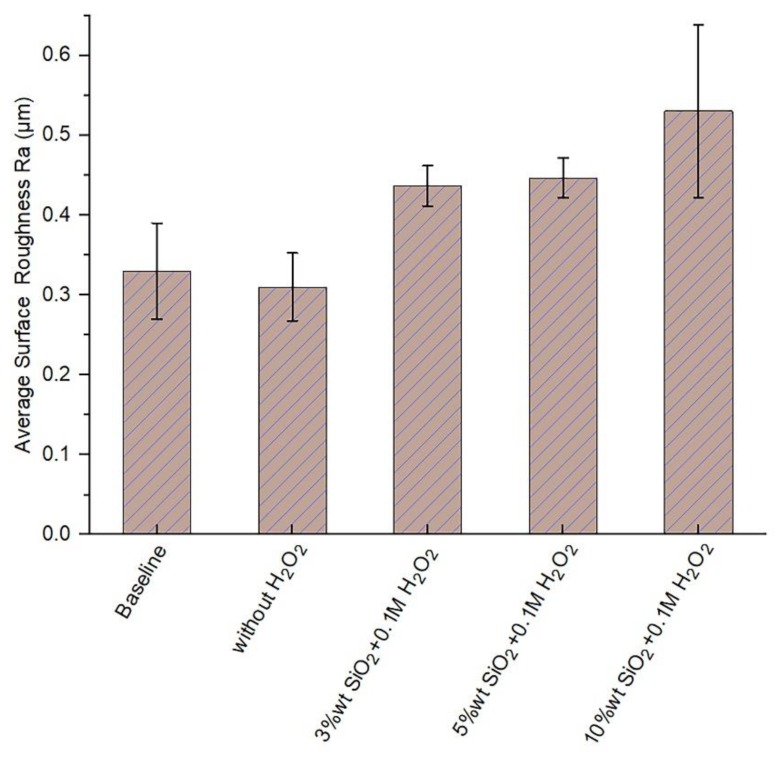
Average surface roughness post 3D CMP (with wool pad) for different SiO_2_ concentrations.

The surface roughness values of the implants polished with the developed process setup are plotted in Figure 7. It can be seen that as the slurry solid loading increased, the surface roughness of the implants also gradually increased. The changes in topographic features had a significant influence on the series of biological events that led to the acceptance of the implant by the host tissue [31], from the adsorption of proteins until the mineralization of the extracellular matrix of the bone tissue, going through the adhesion, proliferation, and differentiation of both osteoblasts and osteoclasts. All these entailed a greater speed in the healing processes and therefore, achievement of faster and biologically more effective osseointegration [32]. Similarly, the use of anodization treatment can allow greater surface roughness, thus diminishing the healing process time associated with the presence of calcium and phosphate ions and promoting bone-implant contact [30].

The interdigitation of the bone tissue with appropriate surface topography effectively reduces the interfacial movement [33]. This factor promotes bone healing in direct contact with the implant and consequently, improves its long-term fixation [34]. This allows the placement of shorter implants, meaning a more manageable implantation procedure is possible for a greater number of surgical situations. For the improvement of implant performance in accordance with the given trends of research, the main strategy followed has been to obtain rougher surfaces of commercially pure titanium (Ti c.p). The preliminary clinical studies of Predecki et al. [35] obtained results that led to a large number of investigations on the relationship between the roughness and healing of the bone tissue. In the specific case of dental implants, an appreciable number of studies have already been carried out to characterize the surface roughness of implants and how they correlate with in vivo implant attachment responses [4,36,37]. In summary, there is sufficient evidence to suggest that roughness improves osseointegration, since

1. Osseointegration occurs more quickly,
2. Higher percentages of bone are in direct contact with the implant, and
3. The resistance to loosening is increased, since higher torques are required for extraction.

However, despite the existence of the mentioned evidence, the fact that corrosion behavior is dependent on surface topography should not be omitted [38].

### 3.4. Potentiodynamic Polarization and Corrosion Rate Calculations

Table 1 shows the potentiodynamic polarization results of Ti-based implants with modified surfaces. Corrosion/passivation data were extracted by using the Tafel extrapolation plots where $I_{corr}$ was calculated through a series of steps involving the Tafel constants *β*_*a*_ and *β*_*c*_, respectively, to calculate the corrosion rate of the sample [39]:

(1) $$I_{corr}=\frac{\beta_{a}\beta_{c}}{2.3\left(\beta_{a}+\beta_{c}\right)}\frac{\Delta I}{\Delta E}$$

The corrosion rate was calculated using the expression [40]

(2) $$Corrosion\;Rate=\frac{0.13\;I_{corr}\left(E.W.\right)}{Density}$$

where *E.W.* is the equivalent weight, $I_{corr}$ is the current density in μA/cm^2^, the density of corroding species in g/cm^3^, and ΔE/ΔI is the slope of the polarization resistance plot, where ∆*E* is expressed in volts and ∆*I* is expressed in μA. The observed corrosion rates were in the order etched > BCP > machined > CMP. The highest $I_{corr}$ was attained with the etched sample, which corresponds to a higher corrosion rate, as illustrated. Higher corrosion rates could indicate higher dissolution rates. Furthermore, in order to correlate the roughness values with the corrosion rates, the results identified from this study were identical to those determined in previous studies [41]. It can be considered that the $I_{corr}$ increases with increased surface roughness [42]. Compared with the high success rate of the anodized Tiunit implants from Nobel Biocare with 1.5 μm roughness, these results demonstrate the ability to adjust the increased surface roughness to diminish the corrosion rate [43]. To prevent peri-implantitis and irritation, the corrosion rate of a metallic implant should be less than 2.5 × 10^−4^ mm/year [44].

### 3.5. Microhardness Test

**Figure 8:**
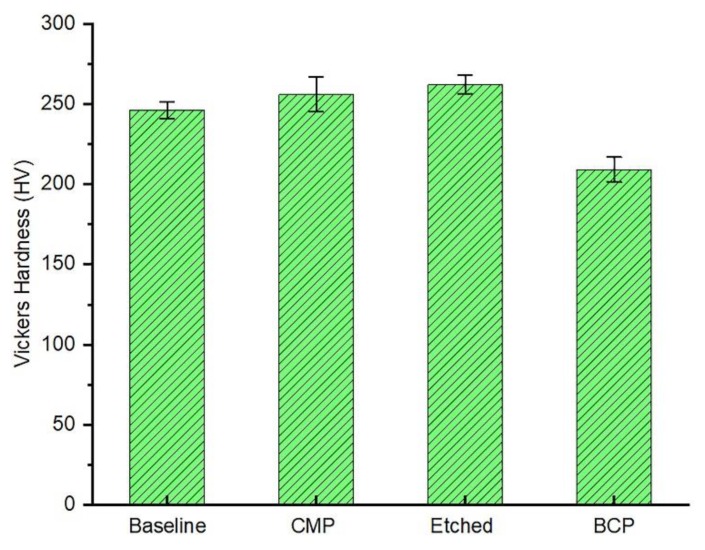
Vickers microhardness test results of the four treated samples.

The results of the microhardness testing of all four samples are shown in Figure 8.

**Figure 9:**
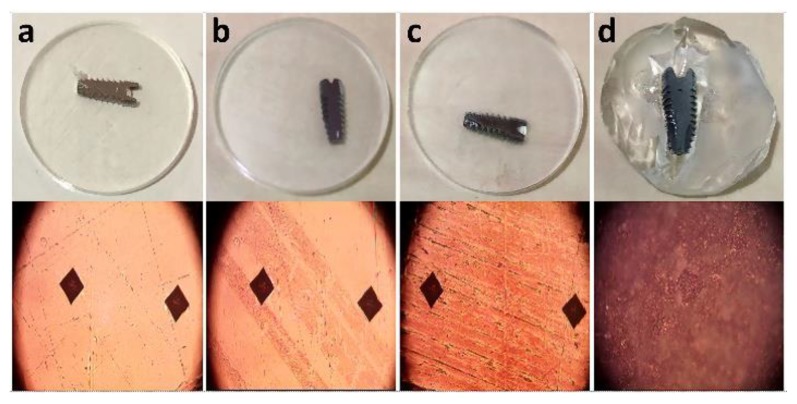
Vickers indentation (50× magnification): (**a**) baseline, (**b**) CMP, (**c**) etched, and (**d**) BCP samples.

Etched Ti-based dental implant samples had greater hardness than the CMP treated samples (2.62 versus 2.56 GPa), and these samples had greater hardness than the BCP treated samples (2.09 GPa). The BCP treated samples were placed under a 1000 g load in order to produce a wide enough indentation that would allow for accurate measurement of the diagonals, as shown in Figure 9. Testing of a 280 HV baseline machined sample provided by the manufacturer of the microhardness testing equipment using the same test procedure and equations used to measure the hardness of the three test samples resulted in a measured hardness of 246 HV. Thus, the hardness measurements reported here are most likely within 0.12% of their actual values. These results emphasize the fact that the stimulation and increase of the titanium oxide layer thickness can enhance the microhardness values due to the high hardness value of TiO_2_. Typically the microhardness results presented by van Vuuren et al. [45] for the anodized dental implants supported the implementation of treatments that induce the production of a thick and uniform oxide layer.

### 3.6. Effect of Surface Topography on the Pull-Out Strength

**Figure 10:**
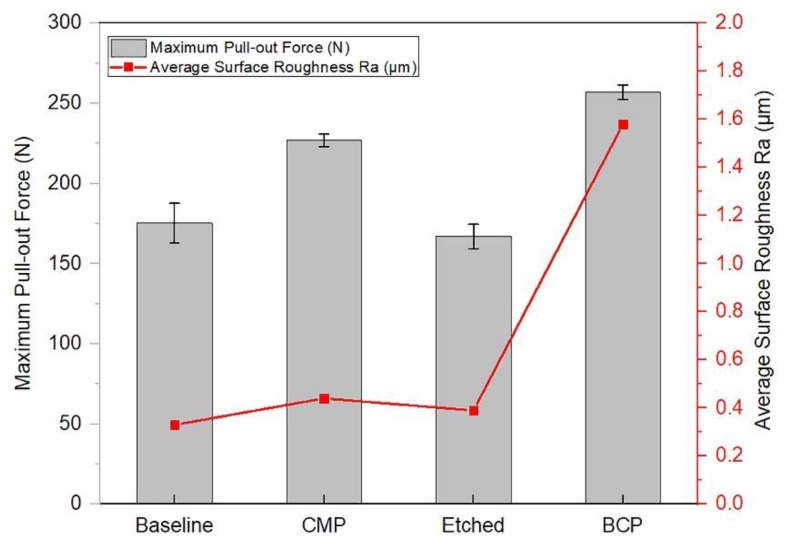
Maximum pull-out force for different surface treatments.

The vertical pull-out strength of each surface treatment was measured in five experiments (Figure 10).

The BCP implants exhibited a greater vertical pull-out strength of 257 N than the CMP (226.8 N) and machined (175.32 N) implants. However, the etched implants had significantly lower strengths than the other surfaces with 167 N. The extracted data demonstrate that relatively rougher implant surfaces increased the bone-to-implant contact tendency and required greater forces to break the bone–implant interface than smoother surfaces. The aim of this test was to evaluate the treated surfaces and to determine if differences exist in the primary stability of implants with relatively smooth surfaces compared to implants with roughened ones.

### 3.7. Removal Torque

**Figure 11:**
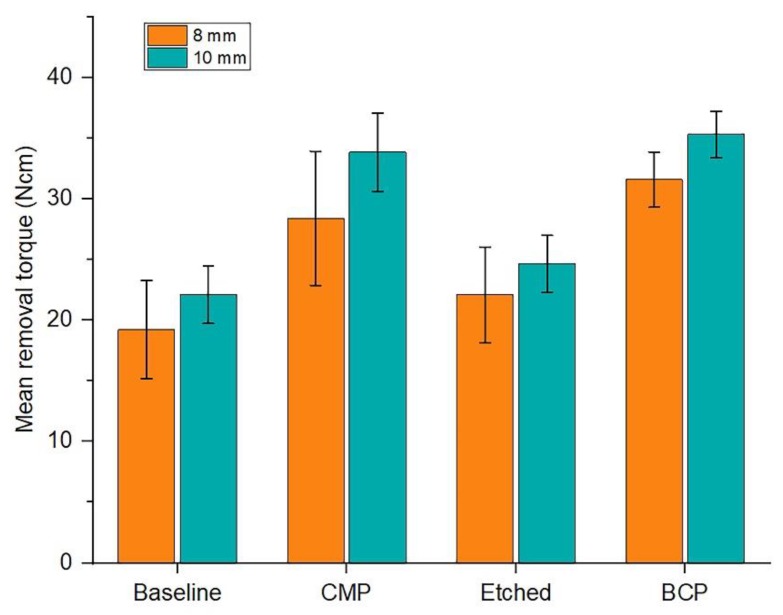
Comparative removal torque mean values between implants measuring 8 and 10 mm in length.

The importance of measuring the implant primary (mechanical) stability at different time points is to allow the evaluation of the long-term implant survival and therefore, the clinical outcome. Therefore, the purpose of this test was to evaluate the implant primary stability of pretreated implants and to give an assessment of the overall success rate of surface-treated dental implants. A secure and successful bone–implant integration is positively associated with the accretion of the contact between the implant and the bone hard tissue, stimulated by implant surface rugosity, which allows for a greater bone–implant adhesion level [46]. Figure 11 illustrates the removal torque test results, which displayed typical values. The peak removal torque was assumed to be the failure torque of the bone–implant interface. The length of the implant and the amount of bone-to-implant contact were the determinants of interface strength in cancellous bone. The results show that the longer implants with 10 mm height gave greater removal torque values due to the larger surface area of the bone-to-implant contact.

**Figure 12:**
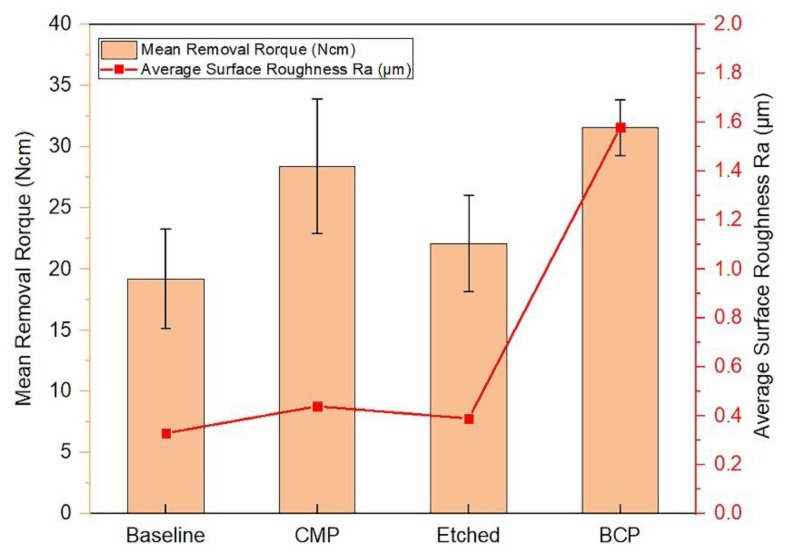
The mean removal torque values for different surface treatments.

Figure 12 shows the influence of the surface roughness on removal torque values in which the CMP treatment increased the implant surface roughness as compared to the machined surfaces. This is one of the fundamental principles for ensuring improved osseointegration of the current surfaces. The data indicate that CMP is a good alternative to commonly implemented implant surface treatments. It produces optimum results that are comparable to those obtained by other surfaces and paves the way for possible clinical application in the context of early and immediate implant loading. In contrast, the results obtained by Koh et al. [47] showed 31.4 Ncm in 2 weeks of insertion of anodized implants which supports the trend taken in this study to modify the bioimplant surfaces in terms of their topography and chemistry.

## 4. Summary and Conclusions

In this study, the 3D CMP process was introduced as an alternative surface structuring technique to engineer the surfaces of cylindrical-threaded dental implants by inducing a controlled surface roughness while stimulating the protective oxide layer forming process. Implementation of 3D CMP with 0.1 M of oxidizer concentration gave the highest passivation on the dental implant surface; these results demonstrated a significant improvement in the corrosion resistance of Ti-based implants that was related to the stimulation of a thin and very uniform Ti-based oxide layer. Similarly, the results showed that the surface roughness and wettability values of the 3D CMP surface treatment enhanced as the contact angle values decreased, which shows that there is an optimal level of surface roughness where a good wettability or hydrophilicity due to high surface energy and small contact angle (less than 90°) occurs. The application of the CMP technique on the Ti-based dental implant pretreated surfaces resulted in similar wettability responses to 2D Ti-based plates, emphasizing that the CMP application can be considered as a type of surface property enhancement for the Ti-based implants. Furthermore, the pull-out and removal torque results also confirmed that the 3D CMP treated surfaces tend to improve the anchorage strengths, thereby increasing the primary stability of Ti-based implants. It is concluded that implementing the 3D CMP treatment prescribed for titanium-based implants enhances the biomechanical anchorage and modifies oxide surface characteristics, therefore improving their bio-corrosion responses.

## Figures and Tables

**Table 1:** Corrosion parameters for the potentiodynamic scans of the titanium-based samples with different surfaces.

Tafel Plot Variables	Baseline	CMP	Etched	BCP
	Titanium Implant Tafel Data			
$I_{corr}$ (μA)	4.52	4.21	13.00	8.93
$E_{corr}$ (mV)	−266.0	−284.0	−381.0	−212.0
Corrosion Rate (mpy)	2.068	1.930	5.933	4.089
Corrosion Rate (mm/year)	0.053	0.049	0.151	0.104
$\beta_{a}$ V/decade	5.435	6.594	7.006	5.974
$\beta_{c}$ V/decade	5.200	4.908	5.465	5.888
